# Oral PrEP use among pregnant or parenting young women in South Africa: evidence from a large community-based implementation study

**DOI:** 10.1186/s12889-026-26370-z

**Published:** 2026-02-09

**Authors:** Jenny Chen-Charles, Dvora Joseph Davey, Elzette Rousseau, Francesca Little, Elona Toska, Ntombovuyo Mathola, Pippa Macdonald, Onesimo Vanto, Melissa Wallace, Linda-Gail Bekker

**Affiliations:** 1 Desmond Tutu HIV Centre, Institute of Infectious Diseases and Molecular Medicine, University of Cape Town, Cape Town, South Africa; 2 Department of Epidemiology and Biostatistics, School of Public Health, University of Cape Town, Cape Town, South Africa; 3 Division of Infectious Diseases, Geffen School of Medicine, University of California, Los Angeles, United States; 4 Department of Statistical Sciences, University of Cape Town, Cape Town, South Africa; 5 Centre for Social Science Research, University of Cape Town, Cape Town, South Africa; 6 Department of Social Policy and Intervention, University of Oxford, Barnett House, 32 -37 Wellington Square, Oxford, OX1 2ER United Kingdom

**Keywords:** Pre-exposure prophylaxis (PrEP), HIV prevention, Adolescent girls and young women, Pregnant and parenting women, LMIC

## Abstract

**Background:**

The risk of HIV acquisition is heightened during pregnancy and early parenthood with the additional risk of vertical HIV transmission. While recent studies have improved our understanding of PrEP use among pregnant and breastfeeding women, further evidence is needed to inform the design of interventions that support sustained use, especially among young women who are pregnant or parenting.

**Methods:**

We analysed data from young women aged 15–29 years who initiated PrEP in an implementation study (FastPrEP) in Cape Town, South Africa. Logistic regression was used to examine the association between pregnancy or parenting status (≥ 1 living child) and PrEP discontinuation at 1- and 4-months post-initiation, based on pharmacy refill data. The primary exposure was currently pregnant or having a child (vs. not); secondary analyses stratified by age (15–24 vs. 25–29 years) among women who were pregnant/parenting. Models were adjusted for age and hypothesised explanatory factors were included in sensitivity analysis: service delivery location, contraceptive use, HIV risk perception, and relationship status.

**Results:**

Between August 2022 and June 2024 *n* = 4,876 young women initiated PrEP; 44% were pregnant/parenting (of which 10% were pregnant), and the median age was 21.6 years (IQR:18–25). At 1-month, women who were pregnant/parenting had higher odds of PrEP discontinuation (aOR:1.30, 95% CI:1.14–1.49). At 4-months this relationship persisted (aOR:1.41, 95% CI:1.12–1.78) compared with non-pregnant/parenting women. Among those pregnant/parenting, younger women (15–24 years) had higher odds of discontinuation at 1-month (aOR:1.31, 95% CI:1.08–1.58) and 4-months (aOR:1.41, 95%CI:1.02–1.96) compared to women aged 25–29. In the fully adjusted multivariable model, receiving PrEP in mobile clinics (aOR:0.71, 95% CI:0.61–0.82) vs. government clinics was associated with lower odds of early discontinuation.

**Conclusion:**

Young women who are pregnant/parenting face elevated risk of early PrEP discontinuation. Differentiated, life-stage and youth-responsive interventions, such as counselling, partner involvement, and integration with maternal and child health, or sexual and reproductive health services, are critical to improving PrEP persistence among this priority population. This population should be prioritised in the rollout of long-acting PrEP formulations, which may better align with their needs and reduce the burden of daily adherence.

**Supplementary Information:**

The online version contains supplementary material available at 10.1186/s12889-026-26370-z.

## Introduction

Adolescent girls and young women (AGYW) aged 15–24 years bear a disproportionate burden of the HIV epidemic in sub-Saharan Africa. In 2023, an estimated 4,000 AGYW acquired HIV each week globally, with approximately 3,100 of these infections occurring in sub-Saharan Africa [1]. AGYW face unique challenges in accessing and adhering to HIV preventive measures, including barriers to health services, gender inequities, and high rates of intimate partner violence [2–4]. There is also increased rates of intentional and unintentional pregnancy among young women in SSA. Furthermore, pregnant and parenting young women face increased biological susceptibility to HIV acquisition, with the additional risk of vertical HIV transmission to offspring, compounded by social and health system barriers [5].

The understanding of pre-exposure prophylaxis (PrEP) initiation and persistence among women, particularly in the context of pregnancy and parenting, is needed to address the HIV epidemic in South Africa and the broader Eastern and Southern Africa region. While suboptimal PrEP uptake and persistence among AGYW in South Africa are well-documented, far less is known about PrEP use specifically among AGYW who are pregnant or parenting [6, 7]. These periods may carry additional stressors that further challenge PrEP use [6–8]. Despite the national rollout of PrEP for pregnant and breastfeeding women since 2021, real-world integration into antenatal and postnatal care settings remains insufficient [9]. Moreover, evidence suggests that while AGYW express interest in PrEP, persistence is low, often interrupted by fears of side effects, perceived stigma, lack of support, or difficulty navigating health systems while also taking on new parenting challenges [10, 11].

Research often treats AGYW, pregnant women, and parenting women as discrete groups. However, young women who are pregnant or new parents need to navigate the overlapping transitions of adolescence, pregnancy, early parenthood simultaneously [12]. These intersecting experiences may shape their HIV prevention needs in unique ways that remain underexplored. While pregnant and parenting women may be motivated by concerns for their own health and wellbeing and that of their unborn or breastfeeding children, younger women may also face distinct social or structural determinants, compared to their older counterparts, that shape their PrEP trajectory [13–15].

This manuscript offers a novel contribution by comparing PrEP use among young pregnant or parenting women aged 15–29 years and their peers who were neither pregnant nor parenting. We further stratify the pregnant/parenting participants by age to evaluate the impact of age on PrEP persistence. By exploring factors such as HIV risk perception, relationship dynamics, and service delivery modality (e.g. mobile versus government clinics), we adopt a holistic approach to understanding the pathways through which pregnancy and parenthood may shape PrEP use [16, 17]. The study draws on real-world data from an ongoing implementation science study in Cape Town, South Africa, an important setting due to its diverse PrEP delivery models and high HIV prevalence. In doing so, we aim to generate insights to inform more tailored PrEP delivery strategies for young women in high HIV prevalence communities navigating pregnancy, parenthood, and HIV prevention.

## Methods

### Study design and setting

The FastPrEP study is an implementation science project conducted in Cape Town, South Africa. The study aimed to evaluate community-wide differentiated PrEP service delivery to scale up the rollout of oral PrEP among adolescents and young people (AYP). Recruitment strategies and more details on study design can be found in the study protocol [18]. Participants initiated PrEP at community-based mobile clinics or local government facilities. During their visit, participants also received counselling and PrEP information. There were four AYP-tailored mobile clinics staffed with a nurse, counsellors specialised in sexual and reproductive health (SRH), and peer navigators (trained young people, 18-29-years-old from the same area). The mobile clinics delivered a range of SRH services, including testing and treatment for HIV and STIs, and pregnancy testing. Therefore, PrEP was delivered through an integrated SRH approach at mobile clinics. Twelve government clinics were part of the study, and existing clinical staff and counsellors provided services. Additionally, two trained peer navigators were allocated to each clinic. Data was collected by trained staff at the sites using REDCap on mobile tablets. This study uses data collected between August 2022 to June 2024.

### Ethics approval and consent to participate

The study received ethics approval from the University of Cape Town Health Sciences Research Ethics Committee (UCT HREC refs: 027/2024; 713/2021) and was conducted in accordance with national and institutional requirements. Informed consent was obtained from all participants. The ethics committee (UCT HREC) approved a waiver of parental or legal guardian consent for adolescents who are minors, aged 15–17 years, so parental or legal guardian consent was not required.

### Study population

The study population comprised of women aged 15–29 years who were eligible and initiated PrEP. Pregnant women were identified through self-report or urine pregnancy test at baseline. Parenting status was determined based on having at least one living child. Among participants classified as pregnant or parenting, a small proportion were pregnant at baseline (10%), with the majority parenting but not pregnant. Pregnant and parenting participants were grouped to reflect the broader perinatal and parenting context for young women. Participants who did not have data on pregnancy or parenting status were excluded from the analysis.

### Outcomes and exposures

The primary outcomes were assessed at one and four months after PrEP initiation and included: (1) PrEP persistence, defined as returning for a refill within 28-days, including a 28-day grace period [19]; (2) PrEP restart, defined as resuming PrEP after missing a scheduled refill; and (3) PrEP discontinuation, defined as not returning for PrEP refills within the persistence window and not restarting.

The primary exposure was pregnancy or parenting status at the time of PrEP initiation. The secondary exposure was age group (youngest women aged 15–24 years vs. women aged 25–29) amongst pregnant/parenting women. Covariates included in the sensitivity analysis include relationship status (in a relationship vs. not), service delivery location (mobile clinic vs. government clinic), and HIV risk perception (moderate-to-high vs. low).

### Analysis

We conducted logistic regression analysis to examine associations between pregnancy or parenting status and all three PrEP outcomes (persistence, restart, and discontinuation) at one month and four months after PrEP initiation.

To inform our analytic approach, we constructed a directed acyclic graph (DAG) to identify a minimally sufficient adjustment set for estimating the total effect of the primary exposures on PrEP discontinuation (see Supplementary Appendix Fig. 1). Based on the DAG, we identified age group (15–24 vs. 25–29) as a potential confounder and included it in all adjusted models.

We conducted sensitivity analyses to examine whether the observed association between pregnancy or parenting and PrEP discontinuation was robust to additional adjustments. Specifically, we tested the inclusion of three theoretically relevant covariates: service delivery model (mobile vs. government clinics), HIV risk perception, and relationship status. These covariates were selected based on theoretical relevance and their plausible influence on both the exposure and the outcome. HIV risk behaviours and testing history at the point of PrEP initiation were included in descriptive analyses to characterise baseline HIV exposure. However, these variables were excluded from the models, as they do not lie on the causal pathway to PrEP discontinuation.

We first estimated unadjusted odds ratios (ORs) for PrEP discontinuation at 1 and 4 months by pregnancy/parenting status. We then fitted a main model adjusting for age, followed by a series of sensitivity models, each adding one covariate at a time, and a final model with all three. All analyses were conducted using STATA version 17. We report ORs and adjusted ORs (aORs) with 95% confidence intervals (CIs).

## Results

Between August 2022 and June 2024, 4,876 young women initiated oral PrEP. The median age was 21.6 years (IQR 18–25), with the majority being younger women aged 15–24 (*n* = 3,583, 73%) and 27% were 25–29 years (*n* = 1,313). Overall, 2,130 participants (44%) were currently pregnant or parenting. Among participants who were pregnant or parenting, 215 (10%) were pregnant at baseline. This included 107 (5%) who were already parenting and 108 (5%) who were pregnant with their first child. More than a third of the youngest women aged 15–24 were pregnant or parenting (34%, *n* = 1166) and almost three quarters (74%, *n* = 965) of 25–29 year olds were pregnant or parenting. Almost a third of participants (29%) had one child, and 13% had more than one child. Among the youngest women aged 15–24, 70% had no children, compared to just 29% of women aged 25–29.

At enrolment, majority reported being in a current relationship (83%), although only 2% were married. Most used mobile clinics to initiate PrEP in the study (73%). Three-quarters had tested for HIV in the past six months, with similar proportions across age groups. Sexual risk behaviours were commonly reported: 13% of participants reported multiple sex partners in the past month, 85% reported inconsistently or not using condoms, and 6% reported symptoms of a sexually transmitted infection (STI) either in themselves or a partner. Self-perceived HIV risk was moderate-to-high among 17% of women (see Table 1).

**Table 1:** Sample characteristics, by age group

	All women who initiated PrEP (*n* = 4876)		
Age	Median = 21.6; IQR = 18–25		
Pregnant or parenting	2125 (44%)		
*Of those who are pregnant or parenting*			
Mothers (not pregnant)	1910 (90%)		
Mothers who are pregnant	107 (5%)		
Pregnant (not mother)	108 (5%)		
Number of living children			
0	2859 (59%)		
1	1394 (29%)		
> 1	622 (13%)		

Factors			
	All women who initiated PrEP (*n* = 4876)	Pregnant or parenting (*n* = 2126)	Not pregnant nor parenting (*n* = 2750)
In a relationship (*n* = 4725) Married	4045 (83%) 97 (2%)	1774 (83%) 85 (4%)	2271 (82%) 12 (0.5%)
Service location Mobile Clinic Government Clinic	3561 (73%) 1315 (27%)	1331 (63%) 794 (37%)	2230 (81%) 521 (19%)
HIV test in past 6 months (*n* = 4859)	3641 (75%)	1626 (77%)	2015 (74%)
Multiple sex partners (≥ 2) (*n* = 4707)	596 (13%)	256 (12%)	340 (13%)
Did not use a condom every time they had sex in last month (*n* = 4783)	4061 (85%)	1713 (87%)	2231 (83%)
Self-reported STI (self or partner) in past month (*n* = 4783)	308 (6%)	165 (8%)	143 (5%)
Moderate-to-high HIV risk perception (*n* = 4784)	830 (17%)	315 (15%)	515 (19%)

### Comparison of PrEP persistence, restart, and discontinuation at 1 and 4 months by pregnancy or parenting status and age groups

Among all women who initiated PrEP (*n* = 4876), 70% discontinued PrEP at 1 month. Discontinuation was higher among pregnant/parenting young women compared to those who were not (72% vs. 68%). In age-adjusted analyses, pregnancy or parenting was associated with higher odds of PrEP discontinuation at 1 month (aOR = 1.30, 95% CI: 1.14–1.49). Pregnant/parenting young women were less likely to persist on PrEP (11% vs. 12%) or to restart PrEP after discontinuation (18% vs. 19%). In age-adjusted analyses, pregnancy or parenting was associated with lower odds of PrEP persistence (aOR = 0.77, 95% CI: 0.64–0.93) or restarting PrEP (aOR = 0.83, 95% CI: 0.71–0.97) at 1 month. While absolute differences were small, these findings suggest early disengagement from PrEP among all young women who were pregnant/parenting (see Table 2).

**Table 2:** PrEP outcomes at 1 and 4 months by pregnancy or parenting status in Cape Town, South Africa (2022–2024)

	Pregnant or parenting women vs. non-pregnant/parenting women			
	Women who are not pregnant/parenting (*n* = 2765)	Women who are pregnant/ parenting (*n* = 2131)	OR (95% CI; *p*-value)	aOR* (95% CI; *p*-value)
PrEP persistence at 1 month	343 (12%; 95% CI: 11–13)	230 (11%; 95% CI: 10–12)	0.85 (0.71–1.01; *p* = 0.071)	0.77 (0.63–0.93; *p* = 0.007)
PrEP restart at 1 month	525 (19%; 95% CI: 18–21)	375 (18%; 95% CI: 16–19)	0.91 (0.78–1.05; *p* = 0.200)	0.83 (0.71–0.97; *p* = 0.022)
PrEP discontinuation at 1 month	1887 (68%; 95% CI: 67–70)	1520 (72%; 95% CI: 70–73)	1.16 (1.02–1.31; *p* = 0.019)	1.30 (1.14–1.49; *p* < 0.001)

*Of those who persisted at 1 month*				
	Women who are not pregnant/ parenting (*n* = 876)	Women who are pregnant and/or parenting (*n* = 610)	OR (95% CI; p-value)	aOR* (95% CI; p-value)
PrEP persistence at 4 months	245 (28%; 95% CI: 26–31)	150 (25%; 95% CI: 22–28)	0.84 (0.66–1.06; *p* = 0.147)	0.77 (0.60-1.00; *p* = 0.054)
PrEP restart at 4 months	130 (15%; 95% CI: 13–18)	81 (13%; 95% CI: 11–16)	0.86 (0.64–1.16; *p* = 0.318)	0.74 (0.54–1.03; *p* = 0.072)
PrEP discontinuation at 4 months	493 (57%; 95% CI: 53–60)	375 (62%; 95% CI: 58–66)	1.24 (1.01–1.54; *p* = 0.044)	1.42 (1.13–1.79; *p* = 0.003)

*adjusted for age

Among the subset of women who persisted or restarted PrEP at 1 month (*n* = 1486), 60% discontinued by 4 months. Discontinuation at 4 months was higher among pregnant/parenting young women compared to their non-pregnant/parenting peers (62% vs. 57%). In age-adjusted analyses, pregnancy or parenting was associated with higher odds of discontinuation at 4 months (aOR = 1.42, 95% CI: 1.13–1.79).

Pregnant/parenting young women were also slightly less likely to persist (25% vs. 28%) or restart PrEP (13% vs. 15%) at 4 months. In age-adjusted analyses, pregnancy or parenting was associated with lower odds of PrEP persistence (aOR = 0.77, 95% CI: 0.60-1.00) and PrEP restart (aOR = 0.74, 95% CI: 0.54–1.03) at 4 months. Although the confidence intervals were wider, the direction of effect consistently pointed toward reduced continuation among pregnant or parenting young women (see Table 2).

When further stratified by age among pregnant/parenting young women, the youngest women aged 15–24 had higher PrEP discontinuation at 1 month compared to women aged 25–29 (74% vs. 69%) and were less likely to restart PrEP at 1 month (16% vs. 20%). In unadjusted analyses, younger age was associated with higher odds of PrEP discontinuation (OR = 1.30, 95% CI: 1.07–1.56) and lower odds of restarting PrEP at one month (OR = 0.79, 95% CI: 0.63–0.98) (see Table 3). These comparisons were unadjusted since age was the stratifying variable.

**Table 3:** PrEP outcomes at 1 and 4 months by pregnancy or parenting status and age in Cape Town, South Africa (2022–2024)

	Youngest women aged 15–24 who are pregnant/parenting vs. women aged 25–29 who are pregnant/parenting		
	Youngest women aged 15–24 who are pregnant/parenting (*n* = 1166)	Women aged 25–29 who are pregnant/parenting (*n* = 965)	OR (95% CI; *p*-value)
PrEP persistence at 1 month	117 (10%; 95% CI: 8–12)	113 (12%; 95% CI: 11–13)	0.84 (0.64–1.10; *p* = 0.202)
PrEP restart at 1 month	187 (16%; 95% CI: 14–18)	188 (20%; 95% CI:18–21)	0.79 (0.63–0.98; *p* = 0.034)
PrEP discontinuation at 1 month	861 (74%; 95% CI: 71–76)	659 (69%; 95% CI: 67–70)	1.30 (1.07–1.56; *p* = 0.008)

*Of those who persisted at 1 month*			
	Youngest women aged 15–24 who are pregnant/parenting (*n* = 305)	Women aged 25–29 who are pregnant/ parenting (*n* = 305)	OR (95% CI; p-value)
PrEP persistence at 4 months	70 (23%; 95% CI: 19–28)	80 ( 27%; 95% CI: 25–30)	0.83 (0.57–1.20; *p* = 0.312)
PrEP restart at 4 months	33 (11%; 95% CI: 8–15)	47 (16%; 95% CI: 13–17)	0.66 (0.41–1.06; *p* = 0.086)
PrEP discontinuation at 4 months	201 (66%; 95% CI: 60–71)	174 (58%; 95% CI: 54–60)	1.42 (1.02–1.98; *p* = 0.036)

Among pregnant/parenting young women, those aged 15–24 had higher PrEP discontinuation at 4 months compared to women aged 25–29 (66% vs. 58%). In unadjusted analyses, younger age was associated with higher odds of PrEP discontinuation (OR = 1.41, 95% CI: 1.02–1.96). However, differences in persistence (23% vs. 27%) and restart (11% vs. 15%) were small. In unadjusted analyses, younger age was not clearly associated with PrEP persistence (OR = 0.83, 95% CI: 0.57–1.20) or restart (OR = 0.67, 95% CI: 0.42–1.08) (see Table 3).

To assess the robustness of our findings and explore potential explanatory pathways for the association between pregnancy/parenting and PrEP discontinuation, we conducted a sensitivity analysis using sequentially adjusted models. We incorporated HIV risk perception, relationship status, and clinic type as theoretically relevant covariates (see Table 4). In the fully adjusted model, which included all three covariates, the association between pregnancy/parenting and discontinuation at 1 month remained (aOR = 1.27, 95% CI: 1.11–1.47). At 4 months, the association was also observed in the fully adjusted model (aOR = 1.39, 95% CI:1.10–1.76). These findings suggest that differences in HIV risk perception, relationship status, or using mobile clinics do not fully account for the increased likelihood of discontinuation among pregnant/parenting young women.

**Table 4:** Analysis of the association between pregnancy or parenting status and oral PrEP discontinuation at 1 and 4 months

Model	Timepoint	Covariate(s) included	Unadjusted OR (95% CI; *p*-value)	aOR (95% CI) (adjusted for age)
1	1 month	None	1.16 (1.02–1.31; *p* = 0.019)	1.30 (1.14–1.49; *p* < 0.001)
2	1 month	Moderate-to-high HIV risk perception	1.17 (1.04–1.33; *p* = 0.012)	1.32 (1.15–1.51; *p* < 0.001)
3	1 month	In a relationship	1.16 (1.03–1.32; *p* = 0.017)	1.30 (1.14–1.49; *p* < 0.001)
4	1 month	Mobile clinic (vs. government clinic)	1.08 (0.95–1.23; *p* = 0.235)	1.21 (1.05–1.39; *p* = 0.007)
5	1 month	All 3 covariates	1.10 (0.97–1.26; *p* = 0.126)	1.24 (1.08–1.42; *p* = 0.002)

*Of those who persisted or restarted at 1 month *(*n *= 1486)				
6	4 months	None	1.24 (1.01–1.54; *p* = 0.044)	1.42 (1.13–1.79; *p* = 0.003)
7	4 months	Moderate-to-high HIV risk perception	1.24 (1.01–1.54; *p* = 0.044)	1.42 (1.13–1.79; *p* = 0.003)
8	4 months	In a relationship	1.24 (1.00-1.53; *p* = 0.051)	1.41 (1.12–1.78; *p* = 0.003)
9	4 months	Mobile clinic (vs. government clinic)	1.22 (0.98–1.51; *p* = 0.072)	1.40 (1.11–1.76; *p* = 0.005)
10	4 months	All 3 covariates	1.22 (0.98–1.51; *p* = 0.070)	1.39 (1.10–1.76; *p* = 0.006)

### Factors associated with PrEP discontinuation

To better understand factors independently associated with discontinuation, we examined the fully adjusted multivariable model including age and all covariates (Appendix Table 1). Receiving PrEP at mobile clinics was associated with lower odds of discontinuation at 1 month (aOR = 0.71, 95% CI: 0.61–0.82), indicating a protective association. Elevated HIV risk perception (aOR = 0.85, 95% CI: 0.72-1.00) and being in a relationship compared to being single (aOR = 0.96, 95% CI: 0.81–1.14) were not strongly associated with discontinuation at 1 month.

At 4-months, none of the covariates were strongly associated with PrEP discontinuation. While the direction of associations remained consistent – for elevated HIV risk perception (aOR = 1.06, 95% CI: 0.81–1.38), receiving PrEP via a mobile clinic (aOR = 0.88, 95% CI: 0.68–1.15), and relationship status (aOR = 0.84, 95% CI: 0.62–1.14).

## Discussion

This study presents novel insights on early PrEP discontinuation among young women who are pregnant or parenting in Cape Town, South Africa. Our findings demonstrate that pregnancy or parenthood among young women was associated with higher odds of PrEP discontinuation, both at one and four months post-initiation, even after adjusting for maternal age. The persistence of this association over time highlights the ongoing challenges this group faces in maintaining PrEP use. While the first month emerged as a particularly high-risk period for disengagement, the elevated risk at four months suggests that sustained support is needed well beyond the point of initiation – a pattern also observed in other studies in the region [10, 14, 20]. Although early PrEP discontinuation was common across all young women initiating PrEP in the cohort, this analysis focused on pregnant or parenting young women to address a specific evidence gap in this population.

To our knowledge, this study is the first implementation science study in sub-Saharan Africa to directly compare PrEP discontinuation patterns between women who are pregnant or parenting and non-pregnant/parenting, using a large sample size and further examining age-related differences between the youngest (15–24 years) and older (25–29 years) women. By examining the role of age, we highlight the heightened vulnerability faced by the very young women (aged 15–24) who are pregnant or parenting, while recognizing that HIV risk remains elevated among women aged 25–29 in this region. These findings contribute new evidence to inform differentiated, age- and context-specific PrEP support strategies, particularly as PrEP delivery expands beyond clinical settings and into more accessible, community-based platforms.

These findings align with prior studies highlighting adherence challenges among AGYW and pregnant or postpartum women, including side effects like nausea and vomiting, which can overlap with pregnancy symptoms and may further complicate adherence, competing priorities, stigma, and limited social support [6, 17–19]. High early PrEP discontinuation has been widely documented among AGYW in implementation settings [10]. The discontinuation observed in this study is consistent with these patterns and may be more clearly characterised by the large sample size and mobile delivery model of this cohort. These findings suggest that early disengagement reflects broader challenges in PrEP delivery rather than pregnancy or parenting alone. Notably, this study demonstrated how the highest adjusted odds of discontinuation were observed at four months among the youngest women (aged 15–24) who were pregnant or parenting, compared to those aged 25–29. This underscores both early and later vulnerability periods for disengagement and the need for sustained support for women who are pregnant or parenting, in particular younger women aged 15–24.

Although we hypothesised that factors such as HIV risk perception and service delivery model (receipt of care in a government clinic vs. mobile clinic) may affect the relationship between pregnancy or parenting and PrEP discontinuation, this was not supported by our analysis. This suggests that these factors do not fully explain the observed relationship. It is possible that other unmeasured or time-varying factors, such as stigma or provider-level barriers, may contribute to early PrEP discontinuation.

The use of mobile clinics in FastPrEP enabled broader access to PrEP and SRH services, particularly for AGYW and those who may not regularly engage with formal health facilities [17]. Within the FastPrEP implementation model, PrEP services were delivered through a differentiated ‘hub-and-spokes’ framework, in which mobile clinics and government clinics served as primary points of initiation and community-based outlets (such as schools, youth clubs, and courier delivery) were intended to support ongoing access and maintenance of PrEP [21]. Our findings reinforce that mobile services are an effective entry point for initiating PrEP, with lower odds of discontinuation observed at one month. However, by four months, mobile clinic users had higher odds of discontinuation. While mobile services support initial engagement, ongoing consistent distribution may require structured follow-up and stronger linkage to healthcare services. In this context, refill-based measures of discontinuation within specific windows may also capture intentional pauses or episodic PrEP use during transitions between service models, rather than permanent disengagement. Complementary participatory research with pregnant or parenting young women from the FastPrEP study has highlighted barriers to sustained PrEP use following initiation, including early clinic closure times, challenges attending clinic appointments, stigma, pill burden, and side effects. Participants identified difficulties navigating clinic-based services after mobile initiation and proposed alternative delivery models, such as extended clinic hours, more mobile and community-based distribution, and long-acting PrEP, as strategies to support continued engagement, which may help explain the attenuation of the mobile clinic protective effect over time [22].

HIV risk perception was not strongly associated with lower odds of discontinuation at one month or four months. Prior studies have shown that HIV risk perception is dynamic and influenced by partner behaviours, trust, and experiences of stigma, factors that may undermine persistence even when initial risk perception is high [16, 23]. Although previous studies have linked relationship status to HIV risk perception and social support, we did not observe a significant association with PrEP discontinuation at either time point [10]. This finding suggests that relationship status alone may not be a reliable predictor of PrEP persistence when other factors, such as pregnancy and age, are considered. Being in a relationship may still increase a sense of HIV vulnerability or reflect better social support – both potential motivators for staying on PrEP [24, 25]. The potential value of engaging partners in PrEP counselling and support strategies, particularly to encourage persistence among AGYW, should therefore continue to be considered in future programming and research [26].

Our findings reinforce the urgent need for differentiated PrEP delivery models, such as the FastPrEP model, that extend beyond initiation and address the specific challenges faced by AGYW, particularly those who are pregnant or parenting. The first month post-initiation emerged as a particularly high-risk period for disengagement, underscoring the importance of anticipatory counselling and tailored support during this early window. Interventions such as early follow-up visits, HIV self-testing, point-of-care STI testing, SMS check-ins, and peer navigation could help mitigate early drop-off [27–32]. However, the continued decline in PrEP persistence observed at 4 months signals that short-term interventions may be insufficient on their own. This later drop-off highlights the need for sustained engagement strategies that respond to evolving needs across the perinatal period. Integrating PrEP with maternal, postnatal, and child health services may further strengthen retention by leveraging existing touchpoints in the health system [6, 9]. As mobile services continue to expand, ensuring smooth and coordinated transitions to ongoing care – whether in community or facility settings – will be essential to support long-term use.

Importantly, this population should be considered and prioritised in the rollout of long-acting PrEP products including injectable PrEP. This study showed alarming drop off at 1 month and then again at 4 months. Long-acting formulations offer extended protection with minimal adherence burden and have demonstrated high efficacy in preventing HIV acquisition among cisgender women [33, 34]. Young women who are pregnant/parenting may benefit substantially from long-acting PrEP as these options would reduce the burden of daily adherence and may be better suited to the realities of young women managing pregnancy, parenting, and other competing priorities, as well as structural barriers to daily oral PrEP. The observed pattern of strong uptake via mobile services but reduced persistence over time suggests that LA-PrEP could be delivered through a staggered model that leverages mobile and community platforms for low-barrier initiation and counselling, followed by less frequent, scheduled LA-PrEP administration through clinic- or community-based services. This approach aligns with emerging evidence that pregnant and breastfeeding women prefer options that minimise refill and clinic visits [35, 36]. Expanding access to long-acting PrEP and embedding it within integrated service platforms could be a critical step toward improving persistence in this high-priority group.

### Strengths and limitations

This study offers new insights into early PrEP discontinuation among pregnant or parenting young women in real-world settings. Because both clinic and mobile service users were included in the large sample size, the results are more widely applicable. By directly comparing discontinuation patterns across age groups and pregnancy/parenting status, the study closes a significant evidence gap in PrEP implementation science. The use of sequentially adjusted models improves the validity of observed associations, and the integration of mobile services reflects the evolving PrEP delivery landscape. These findings provide timely information to inform age- and context-specific and differentiated support strategies for PrEP persistence.

This study has several limitations. First, the study was conducted in a single urban setting and a mobile service delivery model that is not yet widely implemented, which may limit the generalisability of our findings to other settings in South Africa or sub-Saharan Africa. Second, even though our outcome measure used a structured time window to capture discontinuation, it might not capture more subtle patterns of cyclical use or brief pauses in PrEP use. Consequently, some women who were reported as having discontinued PrEP may have done so on purpose, which could result in an overestimation of discontinuation, especially among young women whose use of PrEP may change over time. Third, although we adjusted for age as a key confounder in our models, unmeasured factors such as stigma, or changes in partner dynamics may have influenced PrEP use but were not captured in our data. In addition, information on child age, time since delivery, and breastfeeding status was not collected in this study and therefore could not be examined in the analysis; future studies should collect these data to better understand variation in PrEP persistence across early parenting stages. Fourth, self-reported measures of HIV risk perception may be subject to recall or social desirability bias. Finally, because covariates were only measured at PrEP initiation and not longitudinally, we were unable to assess how changes in individual, relational, or contextual factors over time may have affected PrEP discontinuation at different life stages.

## Conclusions

Young age and pregnancy or parenthood was significantly associated with early PrEP discontinuation at both one month and four months after initiation. This highlights the need for differentiated approaches that address the unique challenges faced by young women during pregnancy and early parenthood, especially the youngest women (aged 15–24). Interventions such as tailored counselling, early follow-up, SMS check-ins, and peer navigation may help mitigate early disengagement. Integrating PrEP with family planning, maternal, and child health services could enhance retention by leveraging existing health system touchpoints. While mobile clinics facilitate initial uptake, ensuring continuity of care through structured follow-up or linkage to facility-based services remains essential. This population, particularly younger women who are pregnant or parenting, should be prioritised in the rollout of long-acting PrEP formulations. Long-acting PrEP formulations may better align with the realities of young women managing pregnancy, parenting, and other competing demands. Embedding long-acting PrEP within integrated, youth-responsive service platforms could be a critical step toward improving persistence and reducing HIV risk in this high-priority group. Our findings contribute novel evidence to inform the design of responsive PrEP delivery models and strengthen HIV prevention efforts for women at heightened risk during pregnancy and early parenthood.

## Supplementary Information

Supplementary Material 1

## Data Availability

The data that support the findings of this study are available from the corresponding author upon reasonable request. The data are not publicly available due to privacy or ethical restrictions.
