# Supplementary material for: Oral PrEP use among pregnant or parenting young women in South Africa: evidence from a large community-based implementation study
Source: BMC Public Health. 2026 Feb 9;26:927. doi: 10.1186/s12889-026-26370-z (PMC13001187; doi:10.1186/s12889-026-26370-z)
Supplement: Supplementary file 1 — Supplementary Material 1 [file 12889_2026_26370_MOESM1_ESM.docx]

**Supplementary Material**

**Appendix Figure 1: DAG**

This DAG illustrates the hypothesized relationships between pregnancy or parenting status at the time of PrEP initiation (exposure) and PrEP discontinuation at 1 and 4 months (outcome). Age group is treated as a confounder, influencing both exposure and outcome. Exploratory covariates—clinic type, relationship status, and HIV risk perception—are included in sensitivity analyses to explore potential explanatory pathways but are not considered confounders.


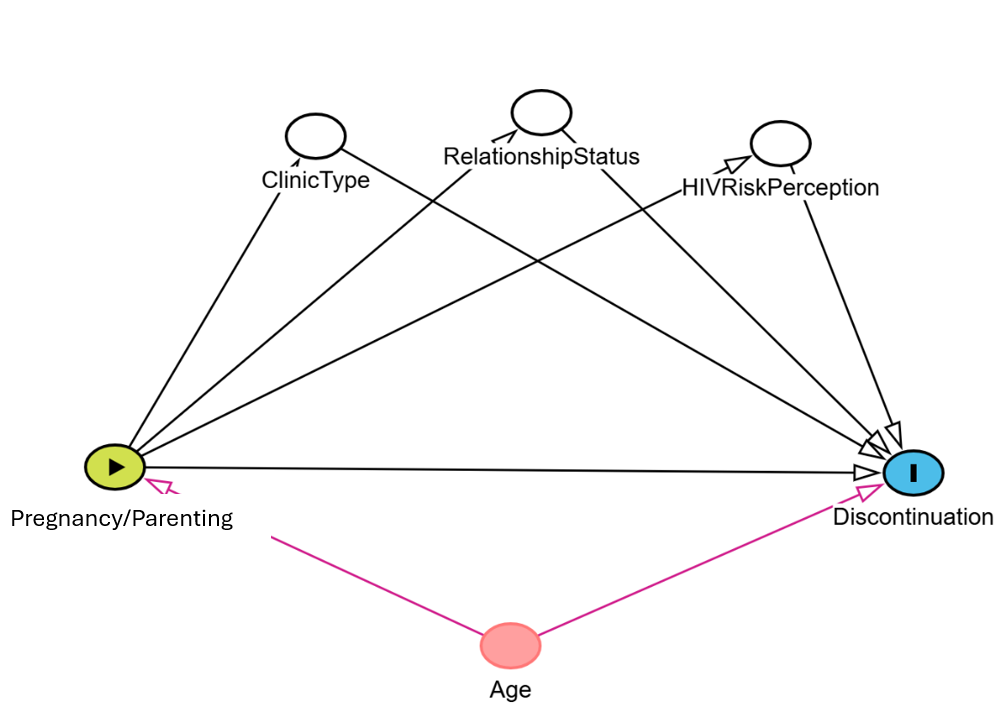


**Appendix Table 1: Full Model including all covariates for Factors Associated with PrEP Discontinuation at 1 and 4 Months**

| **One-month PrEP discontinuation (n=4876)** | | | | |
| --- | --- | --- | --- | --- |
| **Variable** | **N (%)** | **aOR** | **95% CI** | **p-value** |
| Pregnancy or parenthood (vs not pregnant/parenting) | 2125 (44%) | 1.24 | 1.08-1.42 | 0.002 |
| Younger women 15-24 years (vs women aged 25-29) | 3570 (73%) | 1.39 | 1.19-1.61 | <0.001 |
| Moderate-to-high HIV risk perception (vs low) | 830 (17%) | 0.85 | 0.72-1.00 | 0.050 |
| In a relationship (vs not) (n=4725) | 4034 (83%) | 0.96 | 0.81-1.14 | 0.652 |
| Mobile clinic service location (vs gov clinic and others) | 3561 (73%) | 0.71 | 0.61-0.82 | <0.001 |
| **Four-month PrEP discontinuation (n=1474)** | | | | |
| **Variable** |  | **aOR** | **95% CI** | **p-value** |
| Pregnancy or parenthood (vs not pregnant/parenting) | 605 (41%) | 1.39 | 1.10-1.76 | 0.006 |
| Younger women 15-24 years (vs women aged 25-29) | 1032 (70%) | 1.46 | 0.77-1.10 | 0.003 |
| Moderate-to-high HIV risk perception (vs low) | 290 (20%) | 1.06 | 1.02-1.49 | 0.675 |
| In a relationship (vs not) (n=4725) | 1249 (85%) | 0.84 | 0.77-1.16 | 0.272 |
| Mobile clinic service location (vs gov clinic and others) | 1159 (79%) | 0.88 | 1.12-1.58 | 0.348 |
